# Ancestral Sequence Reconstruction for Novel Bifunctional Glutathione Synthetase with Enhanced Thermostability and Catalytic Efficiency

**DOI:** 10.3390/foods15020309

**Published:** 2026-01-15

**Authors:** Jieru Zhao, Binhao Wang, Junhua Di, Jieyu Zhou, Jinjun Dong, Ye Ni, Ruizhi Han

**Affiliations:** 1 Key Laboratory of Industrial Biotechnology, Ministry of Education, School of Biotechnology, Jiangnan University, Wuxi 214122, China; 6230210041@stu.jiangnan.edu.cn (J.Z.); 7220201064@stu.jiangnan.edu.cn (B.W.); 7240201048@stu.jiangnan.edu.cn (J.D.); zhoujieyu@jiangnan.edu.cn (J.Z.); djj0612@jiangnan.edu.cn (J.D.); 2 Institute of Biotechnology, RWTH Aachen University, 52074 Aachen, Germany

**Keywords:** ancestral sequence reconstruction, thermostability, catalytic efficiency, industrial biocatalysis, glutathione, biosynthesis

## Abstract

The bifunctional glutathione synthase (GshF) is able to catalyze glutathione synthesis and is favored for industrial application due to its lack of product inhibition. However, its practical use is limited by moderate catalytic efficiency and poor thermostability. Here, we applied ancestral sequence reconstruction (ASR) to engineer a more robust ancestral GshF (Anc427) with thermal denaturation temperature of 56.2 ± 0.2 °C, representing an increase of 10.8 ± 0.2 °C over the probe enzyme (St-GshF). Additionally, Anc427 exhibited a thermal half-life (t_1/2_) of 3465.7 min at 40 °C, representing a 20-fold increase over that of St-GshF. Under optimal conditions (pH 7.0, 37 °C), Anc427 displayed a specific activity of 3.3 ± 0.02 U·mg^−1^, representing a 20% enhancement compared to St-GshF. Structural modeling and molecular dynamics simulations indicated that the improved stability can be attributed to increased structural rigidity in Anc427. These findings demonstrate that ASR effectively enhances both thermostability and catalytic activity of GshF, significantly advancing its potential for industrial biocatalysis.

## 1. Introduction

Glutathione (GSH), a thiol compound characterized by a γ-peptide bond, is well-known for its defined biological activities, including antioxidant, detoxification, and immunomodulatory functions [1]. Owing to these valuable properties, GSH is extensively utilized in the pharmaceutical, food, and cosmetic industries [2,3,4]. However, the development of efficient and sustainable synthesis routes remains challenging, limiting cost-effective industrial production of GSH [5].

Traditional chemical synthesis of GSH is hampered by significant limitations in efficiency, sustainability, and product quality. For instance, solvent extraction is hindered by its reliance on biological feedstocks, resulting in low productivity and high costs [6]. Furthermore, chemical synthesis generally requires harsh conditions and hazardous reagents, leading to notable environmental risks [7]. Although microbial fermentation remains the predominant industrial approach [8], it is constrained by complex downstream purification processes, modest yields [9,10], and risks of microbial contamination, which restrict its applicability in high-purity settings. Given these inherent limitations, enzymatic synthesis has emerged as a compelling alternative, offering high product specificity [11], mild reaction conditions, and superior environmental compatibility [12].

**Figure 1:**
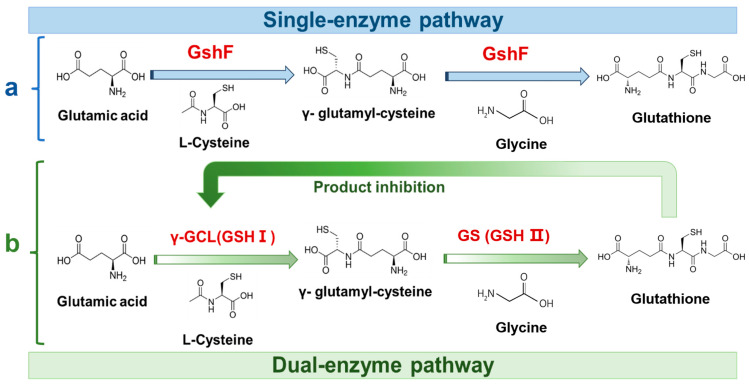
Schematic comparison of glutathione biosynthesis pathways. (**a**) Single-enzyme pathway catalyzed by the fusion enzyme GshF, converting glutamate, cysteine, and glycine into glutathione. (**b**) Dual-enzyme pathway in which γ-glutamyl-cysteine synthetase (γ-GCL, GSH I) and glutathione synthetase (GS, GSH II) sequentially catalyze glutathione formation, with feedback inhibition of γ-GCL by glutathione.

Enzymatic synthesis of GSH has evolved from two-step cascade to more efficient single-enzyme system. The original two-step pathway, involving sequential catalysis by γ-glutamyl-cysteine synthetase (GSH I) and glutathione synthetase (GSH II), was limited by strong feedback inhibition of GSH I by glutathione [13,14]. By integrating the two catalytic functions into one enzyme, bifunctional glutathione synthase (GshF) bypasses the feedback-inhibited route and directly drives ATP-dependent glutathione assembly [15]. This consolidated mechanism (Figure 1) supports efficient ATP regeneration and improves scalability [16]. However, GshF generally exhibits insufficient catalytic activity and poor thermal denaturation temperature (T_m_) (~40–45 °C), restricting its suitability for industrial bioreactors [12,17]. Consequently, protein engineering is required to enhance these essential catalytic properties [18,19].

Strategies for improving GshF performance commonly include site-directed mutagenesis, directed evolution, and rational design [12], each offering distinct advantages and limitations. For instance, site-directed mutagenesis offers simplicity but narrow search radius, typically affording modest improvements such as 5–8 °C increase in T_m_ [20]. Directed evolution couples improvements in stability and activity but is often constrained by extensive screening requirements and long development cycles [21]. Rational design strategies, particularly PROSS-based methods, enable broader multi-point engineering, achieving significant improvements in specific activity (up to 63.62%) and half-life (up to 29.94%) [22]. Nevertheless, their success depends critically on high-resolution structural data and may unintentionally disrupt substrate binding. Representative engineering outcomes include an R270S variant of *S. agalactiae* GshF with a 2.62-fold longer half-life (T_1/2_) at 40 °C [12], a domain-swapped variant displaying 18.16% increase in specific activity together with remarkably extended T_1/2_ at 45 °C [17] and a *S. thermophilus* variant (L136K/V498) exhibiting 86.8% improvement in activity and a 40.95% increase in T_1/2_ [23].

Ancestral sequence reconstruction (ASR) has emerged as a novel protein engineering strategy, leveraging evolutionary information to generate enzymes with significantly enhanced stability. For instance, the T_m_ value of P450s was enhanced by 35 °C using ASR strategy [24,25,26]. The typical ASR workflow follows straightforward pipeline. First, homologous sequences are collected from databases such as UniProt [27] and NCBI [28]. The dataset is then pruned with tools like Treemmer to remove redundant or low-quality entries while preserving phylogenetic signals [29]. Following multiple sequences alignment (e.g., using MEGA [30]), a maximum-likelihood phylogenetic tree is built with algorithms such as IQ-TREE [31]) and assessed for robustness using metrics like the gene concordance factor [32]. Finally, ancestral sequences are inferred with Bayesian methods (e.g., PAML), and high-probability candidates (posterior probability ≥ 0.95) are selected for experimental testing [33].

Given demonstrated strengths of ASR, an optimized evolutionary reconstruction strategy was implemented for GshF in this study. This approach led to the identification of a novel ancestral enzyme, Anc427, which exhibited a 10.8 ± 0.2 °C increase in T_m_ value and a 20 ± 0.1% enhancement in catalytic activity compared with the probe enzyme (GshF from *S. thermophilus*). The resulting enzyme presents a promising scaffold for industrial application and highlights the broader utility of evolutionary information for the rational engineering of bifunctional systems.

## 2. Materials and Methods

### 2.1. Chemicals, Strains, and Plasmids

The probe enzyme GshF was derived from *S. thermophilus* (UniProt ID: Q5M3J8). The codon-optimized gene sequences encoding both the probe enzyme and the ancestral enzyme investigated in this study were synthesized by Yixin Biotechnology Co., Ltd. (Shanghai, China), and cloned into the pET-28a (+) vector between the Bam HI and XhoI restriction sites. Recombinant protein expression was carried out in *Escherichia coli* BL21(DE3). Isopropyl β-d-1-thiogalactopyranoside (IPTG) and kanamycin were sourced from GENERAY (Shanghai, China). A GSH standard was procured from Shanghai Yuanye Bio-Technology Co., Ltd. (Shanghai, China). All other chemicals and reagents were of analytical grade and obtained from Sinopharm Chemical Reagent Co., Ltd. (Shanghai, China).

### 2.2. Ancestral Sequence Reconstruction

ASR was conducted to engineer GshF variants with improved thermal stability, using the *S. thermophilus* GshF (St-GshF) as the probe. The initial step involved retrieving homologous sequences (40–90% identity) from the UniRef90 database via UniProt. To create a high-quality dataset for phylogenetic analysis, sequence redundancy was reduced using Many-against-Many sequence searching (MMseqs2) with a 0.8 similarity cutoff, followed by two rounds of screening to obtain representative sequences. A multiple sequence alignment was generated using Clustal Omega (ClustalW algorithm, version 1.2.4). This alignment provided the foundation for constructing a maximum-likelihood phylogenetic tree in IQ-TREE, where topological confidence was assessed by 1000 bootstrap replicates. The ancestral sequence was then inferred from the tree and alignment using phylogenetic analysis by maximum likelihood, x version (PAML-X).

### 2.3. Protein Expression and Induction

The resulting recombinant plasmids were transformed into *E. coli* BL21(DE3) competent cells using the heat shock method. Transformants were selected on LB agar plates containing 50 μg/mL kanamycin. A single positive colony was inoculated into 10 mL of LB broth (10 g/L peptone, 5 g/L yeast extract and 10 g/L NaCl) with kanamycin (50 μg/mL), then incubated overnight at 37 °C with shaking at 220 rpm to prepare the seed culture. Subsequently, the seed culture was inoculated into 700 mL of TB medium (12 g/L peptone, 24 g/L yeast extract, 0.4% (*v*/*v*) glycerol, 0.017 M KH_2_PO_4_, and 0.072 M K_2_HPO_4_·3H_2_O) supplemented with kanamycin (50 μg/mL). Cells were grown at 37 °C with shaking at 220 rpm until the OD_600_ reached 0.8. To optimize the soluble expression of the target protein, induction was first tested with a range of IPTG concentrations (0.05, 0.1, 0.15, and 0.2 mM) at 25 °C for 12 h, after which optimal expression was achieved by induction with 0.2 mM IPTG under the same conditions. Finally, the cells were harvested by centrifugation (8000× *g*, 5 min) for subsequent purification.

### 2.4. Protein Purification

The cell pellets were resuspended in lysis buffer (20 mM Tris-HCl, 20 mM imidazole, pH 7.4) at a ratio of 0.1 g/mL and lysed on ice via three cycles of high-pressure homogenization. Then the lysate was centrifuged at 8000× *g* for 30 min at 4 °C. The supernatant was collected and filtered through a 0.22 μm membrane to obtain the crude enzyme extract. Proteins purification was performed using an AKTA pure chromatography system (AKTA) using a 5 mL His Trap HP column from Qianchun Biotechnology Co., Ltd. (Yancheng, China). The mobile phases consisted of buffer A (20 mM imidazole, 20 mM Tris-HCl, pH 7.4), buffer B (500 mM imidazole, 20 mM Tris-HCl, pH 7.4), and buffer C (100 mM Tris-HCl, pH 7.4). The nickel-charged affinity column was sequentially equilibrated with ultrapure water and buffer A. After loading the crude extract, the column was washed with two column volumes of buffer A to remove unbound proteins. The target protein was then eluted using a linear gradient of buffer B (0–100%). Elution fractions were collected automatically with an absorbance threshold set at 50 mAU. Fractions containing the target protein, as identified by sodium dodecyl sulfate–polyacrylamide gel electrophoresis (SDS-PAGE) analysis showing a single band at the expected molecular weight, were pooled and concentrated to approximately 2 mL using an ultrafiltration centrifugal device with 30 kDa molecular weight cut-off. Imidazole was removed from the concentrated protein solution via buffer exchange into 100 mM Tris-HCl (tris(hydroxymethyl)aminomethane hydrochloride, pH 7.4) using a pre-equilibrated desalting column. The final purified protein was concentrated to 2 mg/mL, as determined by a Nano Drop 2000 spectrophotometer, flash-frozen in liquid nitrogen, and stored at −80 °C for subsequent use.

### 2.5. Enzyme Activity Assay

The enzymatic assay was performed using the purified enzyme. Activity was determined in a 100 μL reaction mixture containing 100 mM Tris-HCl (pH 7.0), 20 mM MgCl_2_, 20 mM l-glycine, 20 mM l-glutamate, 20 mM l-cysteine, and 20 mM ATP. The mixture was pre-incubated at 37 °C for 2 min, and the reaction initiated by adding 10 μL of the enzyme solution. The reaction was then carried out at 37 °C with shaking at 800 rpm for 10 min and was terminated by adding an equal volume of 25% (*w*/*v*) trichloroacetic acid (TCA). After vortex mixing, the terminated reaction was kept on ice for 30 min and then centrifuged at 12,000× *g* for 10 min at 4 °C. The resulting supernatant was collected, diluted 10-fold with an appropriate solvent, filtered through a microporous membrane, and subjected to GSH quantification. One unit of enzyme activity (U) was defined as the amount of enzyme required to catalyze the formation of 1 μmol of GSH per minute under the assay conditions.

### 2.6. GSH Content Analysis

#### 2.6.1. DTNB Spectrophotometric Assay

The concentration of reduced GSH was quantified using a DTNB-based spectrophotometric method [34]. This assay relies on the reaction of DTNB with thiol groups to yield a yellow-colored product with an absorption maximum at 412 nm. To eliminate interference from free cysteine and other thiol-containing compounds, formaldehyde was introduced to selectively mask non-GSH thiols. Briefly, a DTNB stock solution (10 mM) was prepared in 50 mM disodium hydrogen phosphate buffer (pH 7.0). A working solution (0.1 mM) was obtained by a 100-fold dilution of the stock with 0.25 M Tris-HCl buffer (pH 8.5). In a 96-well plate, the sample solution, 0.15 M NaOH, and 3% formaldehyde were combined in a 1:3:1 volume ratio. After incubation for 2 min, five volumes of the DTNB working solution were added. The reaction proceeded at 25 °C for 5 min before the absorbance was measured at 412 nm. The GSH content in samples was determined by interpolation from a standard curve (Figure S1 and Table S1).

#### 2.6.2. High-Performance Liquid Chromatography (HPLC) Analysis

GSH content was additionally quantified by HPLC. Separation was performed on a Diamonsil C18 column (5 μm, 250 mm × 4.6 mm) maintained at 30 °C. The mobile phase consisted of a 95:5 (*v*/*v*) mixture of aqueous solution and acetonitrile. The aqueous solution contained 0.1 mol/L potassium dihydrogen phosphate and 0.01 mol/L sodium 1-heptanesulfonate, with pH adjusted to 2.65. Isocratic elution was carried out at a flow rate of 0.8 mL/min. Detection was performed using a UV detector set at 210 nm. The injection volume was 10 μL. Quantification was based on an external standard curve (Figure S1).

### 2.7. Optimization of Enzymatic Reaction Conditions

The optimal reaction conditions for the ancestral enzyme were determined by systematically evaluating four key parameters: pH, temperature, enzyme concentration, and reaction time. The effect of pH was assessed using PBS (phosphate-buffered saline, 100 mM, pH 6.0–8.0) and Tris-HCl (100 mM, pH 7.0–9.0) buffers. Following the identification of the optimal pH, the temperature dependence of the enzyme activity was examined across a range of 20 °C to 60 °C. Subsequently, the enzyme concentration was varied from 0.02 to 0.3 mg/mL, and the reaction time course was monitored over 60 min. With the exception of the time-course experiments, all reactions were terminated after 10 min of incubation. Enzyme activity in all assays was determined by quantifying GSH production using the DTNB method.

### 2.8. Thermostability Analysis

The thermostability of the purified enzyme was characterized using two complementary approaches: thermal inactivation kinetics and calorimetric analysis of protein unfolding.

Thermal Inactivation Kinetics: T_1/2_ was determined by incubating the enzyme solution (1 mg/mL) at 40 °C and 50 °C. At predetermined times, samples were withdrawn, rapidly cooled on ice to arrest inactivation, and then assayed for residual catalytic activity under the standard assay conditions (37 °C, 10 min; DTNB method). Residual activity was expressed as a percentage relative to a non-heated control (defined as 100%). T_1/2_ at each temperature was derived as the time point at which the activity decreased to 50%.

Calorimetric Analysis: Tₘ was measured by differential scanning calorimetry (DSC). The enzyme was prepared at a concentration of 3 mg/mL in Tris-HCl buffer (pH 7.4). Thermal denaturation was monitored using a Nano-DSC instrument with a temperature ramp from 20 °C to 80 °C at a rate of 1 °C/min. The resultant heat flow curve was analyzed using the Two-State Scaled model within the Nano Analyze™ software (version 3.4.0), and the Tₘ was identified as the temperature corresponding to the maximum of the endothermic peak.

### 2.9. Reaction with Elevated Concentration Substrate

The yield of GSH was evaluated in a 50 mL reaction mixture containing 100 mM each of cysteine, glutamate, glycine, MgCl_2_, and ATP, with the purified enzyme added to a final concentration of 0.06 mg/mL (pH 7.0). The temperature was maintained at 37 °C throughout to enable consistent comparison of catalytic performance. To prevent acidification due to phosphate release during the reaction, the pH was stabilized at 7.0 via automatic titration with 1 M NaOH, thereby maintaining optimal activity of this neutral-pH-active enzyme.

Unlike the 10 min activity assay, which shared an otherwise identical composition, this yield assay was conducted over an extended period. During the reaction, 1 mL aliquots were collected at predetermined time intervals. Each aliquot was immediately quenched with an equal volume of 25% (*w*/*v*) trichloroacetic acid (TCA), followed by a 50-fold dilution to eliminate interference from TCA in the HPLC analysis prior to product quantification.

### 2.10. Steady-State Kinetic Analysis

Steady-state kinetic parameters were determined by varying the concentration of cysteine, a key substrate in glutathione biosynthesis [35], while maintaining the concentrations of the other substrates at saturating levels.

Initial reaction rates were measured under the same conditions as the standard enzyme activity assay (37 °C, 10 min; DTNB method). The substrate concentration range of cysteine was 2–30 mM. Kinetic parameters, including the Michaelis constant (*K*_m_) and turnover number (*k*_cat_), were obtained by nonlinear regression fitting of the initial rate data to the Michaelis–Menten equation using Origin (version 2022). Each experiment was performed in triplicate.

### 2.11. Molecular Dynamics (MD) Simulations

The crystal structures of St-GshF and the ancestral enzyme were predicted using AlphaFold2 (DeepMind, London, UK), and the model quality was evaluated with SAVES v6.1 (Structure Analysis and Verification Server, UCLA, USA), with the best model selected for subsequent MD simulations. Hydrogen atoms were added using the H++ server. MD simulations were performed with GROMACS 2023 software package for 40 ns using the Amber99sb-ildn force field and TIP3P water model. The protein was solvated in a dodecahedral box with a minimum distance of 10 Å from the box edge. Na^+^ or Cl^−^ ions were added to neutralize the system. Energy minimization was followed by equilibration in NVT and NPT ensembles for 100 ps each. Production simulations were run for 40 ns with a 2-fs time step.

## 3. Results and Discussion

### 3.1. Ancestral Sequence Reconstruction for GshF

**Figure 2:**
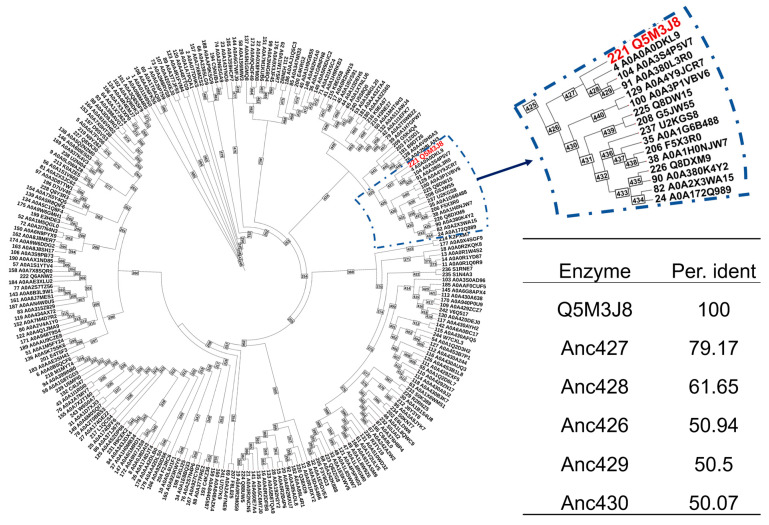
Ancestral sequence reconstruction of GshF. The analysis generated 242 reconstructed protein sequences (IDs 245–486, shown in purple) representing key evolutionary intermediates at major phylogenetic nodes, along with 244 sequences retrieved from databases (IDs 1–244). The red node indicates the UniProt accession number of the probe enzyme. The table presents a sequence identity comparison between the reconstructed ancestral enzymes and St-GshF, with the top five ancestral sequences showing the highest sequence identity listed.

To enhance enzyme thermostability, ASR was performed using the thermostable enzyme St-GshF (from *S. thermophilus*) previously reported as the probe [17], yielding 242 putative ancestral proteins representing key intermediates at major phylogenetic nodes (Figure 2). The reconstruction procedure effectively removed redundant sequences while retaining essential evolutionary signals. A reliable phylogenetic framework was obtained from Clustal Omega and a maximum-likelihood phylogenetic tree generated with IQ-TREE, with all major branches supported by bootstrap values above 85%. This robust phylogeny provided a solid foundation for subsequent ancestral inference using PAML-X. To prioritize candidates for experimental evaluation, ancestral sequences sharing at least 50% sequence identity with the probe enzyme were selected, yielding the top five candidate ancestral enzymes (Figure 2, table). Among these candidates, Anc427 was selected for further characterization because of its closest evolutionary proximity to the probe enzyme, which is expected to minimize functional divergence while retaining ancestral features. The full-length amino acid sequence of Anc427 is provided in Table S2.

### 3.2. Heterologous Expression and Purification of the Ancestral Enzyme

**Figure 3:**
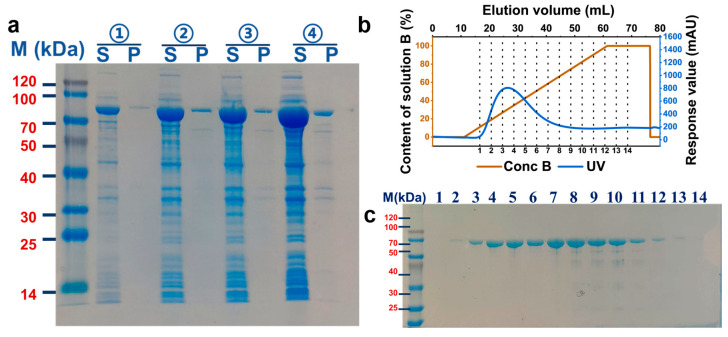
Optimization of expression and affinity purification of the Anc427. (**a**) SDS-PAGE analysis of protein expression under different IPTG concentrations. Lanes: M, molecular weight marker; S, soluble fraction; P, insoluble fraction. IPTG concentrations: ① 0.05 mM, ② 0.1 mM, ③ 0.15 mM, and ④ 0.2 mM. (**b**) AKTA purification profile. Blue: UV absorbance trace; brown: elution buffer gradient (500 mM imidazole). (**c**) SDS-PAGE analysis of affinity-purified fractions. Fractions 1–14 with absorbance above 50 mAU show a single band at the expected molecular weight, indicating high purification efficiency.

To investigate subsequent biochemical characterization, Anc427 was heterologously expressed in *E. coli* BL21 (DE3) and purified. Optimization of induction conditions identified 0.2 mM IPTG as optimal concentration for obtaining soluble protein in the supernatant (Figure 3a), suggesting an effective balance between protein production and host cell viability. SDS-PAGE analysis confirmed robust expression of the target protein, showing predominant band at approximately 85 kDa, consistent with the theoretical molecular weight of 85.06 kDa. The optimized condition was subsequently used for Anc427 expression, yielding preparations of sufficient purity for downstream functional analyses.

Affinity purification of Anc427 was carried out using a 5mL His Trap HP column on AKTA system, with the protein eluted using imidazole gradient of 300–500 mM (Figure 3b,c). Fractions exceeding an automated threshold of 50 mAU were selectively collected, minimizing nonspecific contaminants. SDS-PAGE analysis of these fractions revealed a single band at expected molecular weight, indicating high purity. The pooled protein was subsequently concentrated to 2 mg/mL, producing material suitable for detailed enzymatic characterization. This expression-purification workflow provided a robust and reproducible basis for subsequent biochemical analysis.

### 3.3. Characterization of the Ancestral and Probe Enzymes

**Figure 4:**
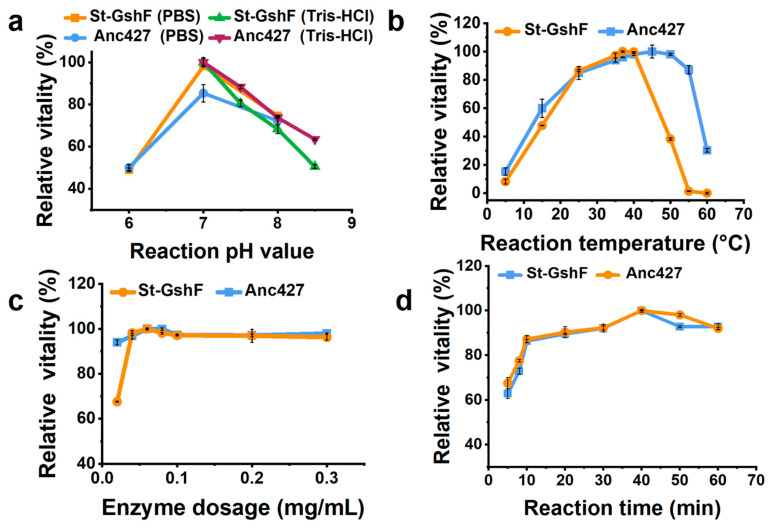
Optimization of reaction conditions for Anc427. Effects of (**a**) pH, (**b**) temperature, (**c**) enzyme concentration, and (**d**) reaction time on relative activity.

After purification, the catalytic properties of Anc427 and St-GshF were characterized (Figure 4). As shown in Figure 4a, the effect of pH on enzymatic activity was analyzed. Both Anc427 and St-GshF exhibited maximal activity at pH 7.0 in Tris–HCl, and comparable activity was observed in PBS at the same pH. Anc427 retained more than 80% of its relative activity between pH 6.0 and 8.0, indicating effective operation within neutral range conditions. This narrow yet efficient pH profile suggests that the ionization state of key catalytic residues is critical for catalysis, consistent with adaptation to near-neutral cytosolic conditions.

Effect of reaction temperature on enzymatic activity was subsequently assessed (Figure 4b). Anc427 displayed maximal activity at approximately 45 °C and sustained high catalytic output across 35–50 °C. In contrast, St-GshF exhibited optimal activity at 37 °C, with a sharp decline observed above 40 °C. Notably, only 40% of its activity was retained at 50 °C. To enable a consistent comparison between the two enzymes, 37 °C was selected as the standard reaction temperature, as it remained within the functional range of both enzymes, supported high catalytic efficiency, and facilitated reproducibility. The broader thermal profile of Anc427 indicated enhanced structural robustness under elevated temperatures.

Enzyme concentration was then optimized across a range of 0.02–0.3 mg/mL (Figure 4c). Activity increased with concentration and plateaued at 0.06 mg/mL, which was therefore selected to maintain proportionality between enzyme amount and reaction rate. Reaction time was evaluated from 5 to 60 min (Figure 4d). Product formation remained linear within the first 10 min for both enzymes, establishing this period as appropriate for initial-rate measurements.

Based on these results, the standardized assay conditions were defined as pH 7.0, 37 °C, an enzyme concentration of 0.06 mg/mL, and a reaction time of 10 min. These conditions provide a consistent basis for subsequent comparisons of catalytic efficiency and substrate turnover.

### 3.4. Thermostability of the Ancestral Enzyme and Probe Enzymes

**Figure 5:**
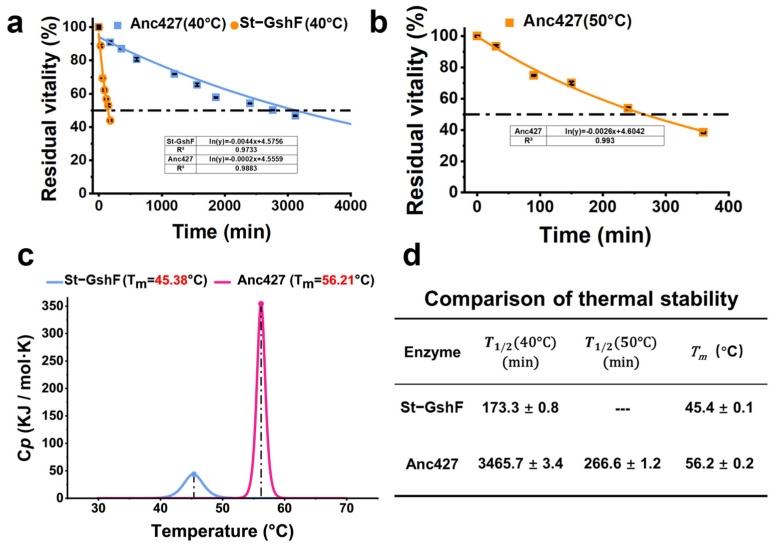
Thermostability investigation of Anc427 and St-GshF. (**a**) Residual activity decay curves at 40 °C. (**b**) Residual activity decay curves at 50 °C. St-GshF is completely inactivated at 50 °C. The dashed lines in (**a**,**b**) indicate the 50% residual activity threshold for T_1/2_ determination. (**c**) Thermal denaturation profiles measured by differential scanning calorimetry (DSC), showing thermal denaturation temperature (Tₘ). (**d**) Summary of key stability parameters: T_1/2_ at 40 °C, T_1/2_ at 50 °C, and Tₘ. In panels (**a**,**b**), the blue and orange curves represent St-GshF and Anc427, respectively. In panel (**c**), the blue and rose red curves represent St-GshF and Anc427, respectively.

Subsequently, the thermostability of Anc427 and St-GshF was evaluated (Figure 5). Consistent with optimal-temperature assays (Figure 4b), Anc427 exhibited significantly enhanced resistance to thermal inactivation. At 40 °C, Anc427 showed an extended T_1/2_ (~3465.7 min) compared with that of St-GshF (~173.3 min) (Figure 5a,d). At 50 °C, Anc427 retained a T_1/2_ of approximately 266.6 min, whereas St-GshF was completely inactivated under the same conditions (Figure 5b,d), indicating a substantial improvement in operational stability of the ancestral enzyme compared with the probe enzyme.

To further investigate the structural basis of these differences, thermal denaturation was analyzed by differential scanning calorimetry (DSC) (Figure 5c). St-GshF displayed a T_m_ of 45.4 ± 0.1 °C, while Anc427 exhibited a substantially higher Tₘ of 56.2 ± 0.2 °C. This increase of nearly 11 °C provided thermodynamic evidence for enhanced structural rigidity of the ancestral enzyme, consistent with its superior performance in functional stability assays. Collectively, these findings clearly establish Anc427 as a significantly more thermostable biocatalyst than the probe enzymes.

### 3.5. High-Substrate Concentration Reaction

**Figure 6:**
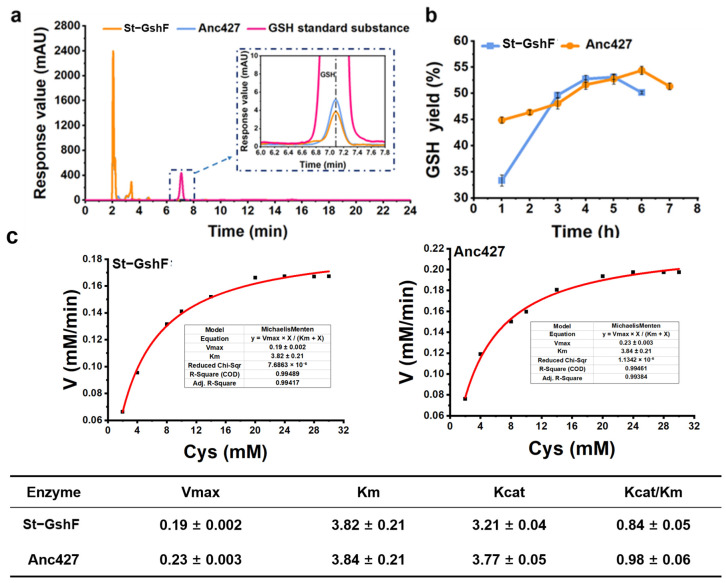
Enzymatic activity, time-course yield profile, and steady-state kinetic parameters of the ancestral enzyme Anc427 and St-GshF under scaled-up reaction conditions. (**a**) HPLC profiles for the enzymatic reaction. The rose red, orange, and blue curves correspond to the GSH standard, St-GshF, and Anc427, respectively. (**b**) Yield of GSH synthesis in a 50 mL reaction system containing 100 mM substrate. Data points represent the mean ± SD from three independent replicates. (**c**) Steady-state kinetic parameters of St-GshF and the ancestral enzyme Anc427 determined using cysteine as the variable substrate. The apparent Michaelis–Menten parameters, including V_max_, *K*_m_, *k*_cat_, and catalytic efficiency (*k*_cat_/*K*_m_), were obtained by nonlinear regression fitting to the Michaelis–Menten equation. Data are presented as mean ± standard deviation from three independent experiments.

To evaluate the catalytic performance of Anc427 under conditions resembling industrial applications, a scaled-up reaction system was employed (Figure 6). In the enlarged-volume assay, Anc427 exhibited a specific activity of 3.3 ± 0.02 U·mg^−1^, which was higher than that of St-GshF (2.81 ± 0.03 U·mg^−1^) (Figure 6a). This enhanced activity under process-relevant conditions indicated that the ancestral enzyme retains high catalytic efficiency when transitioning from analytical to preparative scale.

Time-course analysis of glutathione formation further highlighted the performance advantage for Anc427 (Figure 6b). After 6 h of reaction, Anc427 achieved a yield of 54.4 ± 0.2%, corresponding to a product concentration of 16.7 ± 0.3 g·L^−1^. In contrast, St-GshF reached a maximum yield of only 53.1 ± 0.4% at 5 h, after which no further product accumulation was observed. Notably, Anc427 continued to catalyze glutathione production beyond 5 h, demonstrating sustained catalytic activity and enhanced operational stability under scaled-up conditions.

Despite these advances, the overall yield remained below 80%, suggesting that product accumulation is now limited by ATP supply rather than catalytic turnover. Future work may focus on integrating ATP-regeneration modules, such as polyphosphate kinase systems or multi-enzyme ATP-replenishing cascades, to enable higher substrate yield and sustained reaction performance at scale.

To further elucidate the intrinsic catalytic mechanism of both St-GshF and Anc427 for improved performance at scale, we measured the kinetics parameters using cysteine as the variable substrate (Figure 6c) [35]. Consistent with the scaled-up results, Anc427 showed a higher turnover number (*k*_cat_) and catalytic efficiency (*k*_cat_/*K*_m_) than St-GshF, with comparable substrate affinity. This indicates that Anc427 has enhanced catalytic capacity without losing its ability to bind substrate.

Overall, the higher specific activity, extended catalytic persistence, and enhanced productivity under prolonged reaction conditions collectively establish Anc427 as a promising biocatalyst for industrial processes that require robust performance at the preparative scale.

#### Structural Modeling and MD Simulations

**Figure 7:**
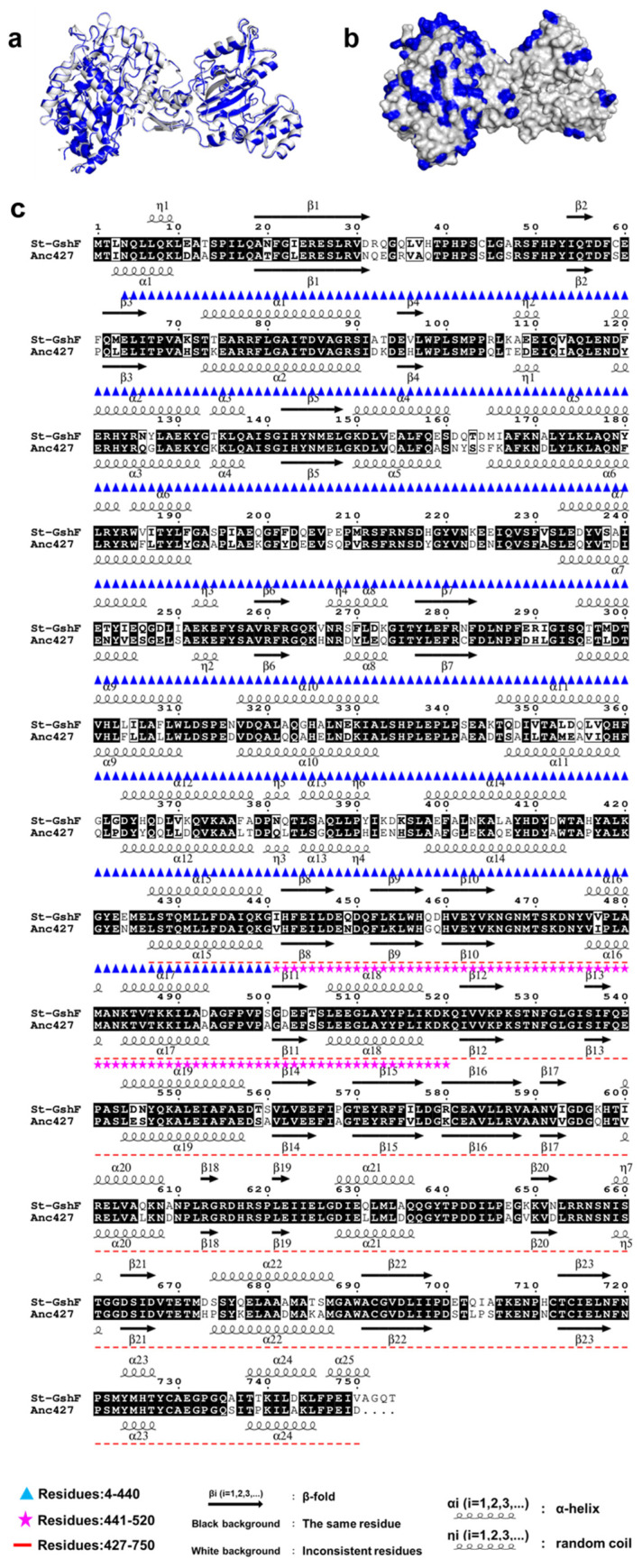
Structural prediction and sequence alignment. (**a**) Structural alignment of St-GhF and Anc427. St-GhF is shown in gray, and Anc427 in blue. The three-dimensional structures of both were predicted using AlphaFold2. (**b**) Schematic structure of Anc427. Residues that differ from St-GhF after sequence alignment are highlighted in blue. (**c**) Sequence alignment performed using Esprit 3.0 [36,37,38,39,40].

The three-dimensional structures of Anc427 and St-GshF were predicted using AlphaFold2, revealing a striking structural conservation between the two enzymes (Figure 7a). Anc427 retains the canonical two-domain architecture as well as the conserved catalytic machinery of St-GshF, including identical active-site residues and substrate-binding motifs, consistent with the known substrate-channeling mechanism of GshF family [16]. This pronounced structural conservation strongly suggests that Anc427 possesses an intact catalytic core comparable to that of its modern counterpart, thereby providing a structural basis for its anticipated enzymatic activity. Consequently, the enhanced thermostability of Anc427 is unlikely to arise from alterations within the catalytic core region. Moreover, sequence alignment indicates that Anc427 shares 71% sequence identity with St-GshF, with most of the non-conserved residues located on the surface of the Anc427 structure (Figure 7b,c). This distribution implies that these surface substitutions may contribute to the increased structural rigidity observed in Anc427. Notably, Anc427 showed a reduction in disordered and coil-like elements compared to St-GshF (Figure 7c), suggesting a more rigid and cooperatively stabilized protein scaffold.

**Figure 8:**
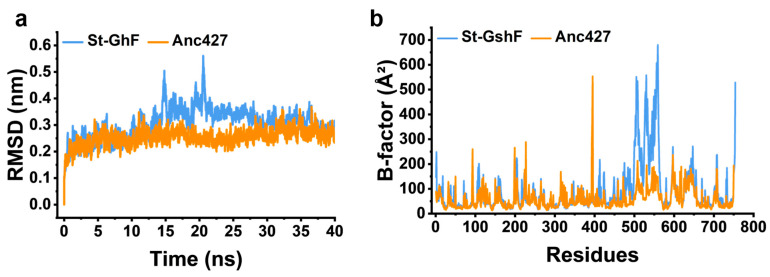
Molecular dynamics simulation analysis. (**a**) Root mean square deviation (RMSD) of the protein backbone, reflecting the overall structural fluctuation. (**b**) Per-residue B-factor distribution, indicating local conformational flexibility. In both panels, the blue and orange curves correspond to St-GshF and Anc427, respectively.

Furthermore, MD simulations were performed (Figure 8) to validate the increased structural rigidity of Anc427 at the atomic level. Root mean square deviation (RMSD) analysis, reflecting global backbone fluctuations, showed significantly lower values of Anc427 than St-GshF (Figure 8a), suggesting the enhanced conformational stability of Anc427. Correspondingly, per-residue B-factor analysis revealed a globally suppressed flexibility profile in Anc427 (Figure 8b). Notably, the B-factor of the region comprising residues 500–550 displayed significant decline in Anc427 compared to St-GshF (Figure 8b), suggesting reduced flexibility and enhanced rigidity of Anc427 structure. This indicates that distal mutations introduced during ASR exert allosteric effects, rigidifying this flexible loop and collectively contributing to the enzyme’s improved thermostability. The stabilization of such dynamically sensitive regions, arising from the long-range effects of ancestral substitutions, provides a coherent mechanistic explanation for the superior thermostability of Anc427.

## 4. Conclusions

The industrial synthesis of glutathione is constrained primarily by the limited activity and insufficient stability of existing biocatalysts. By ASR, this study established an evolution-guided framework for engineering GshF and generated a variant with substantially improved properties. The engineered enzyme achieved a >10 °C increase in T_m_ and specific activity of 3.3 ± 0.02 U·mg^−1^, demonstrating that thermostability and catalytic efficiency can be simultaneously enhanced through ASR.

Overall, this work delivers not only a promising biocatalyst for industrial glutathione production but also a compelling demonstration of ASR as a practical and effective strategy for resolving stability–activity trade-offs in complex, multifunctional enzymes. The methodological framework established here is broadly applicable and offers a generalizable route toward the rational enhancement of industrial biocatalysts.

## Data Availability

The original contributions presented in the study are included in the article/Supplementary Materials. Further inquiries can be directed to the corresponding authors.

## Supplementary Materials

The following supporting information can be downloaded at: https://www.mdpi.com/article/10.3390/foods15020309/s1, Table S1: Reaction system for glutathione (GSH) determination using the DTNB method; Table S2: Sequence identity comparison between the reconstructed ancestral enzymes and the probe enzyme (St-GshF); Table S3: The amino acid sequence of Anc427; Figure S1: Standard curve for reduced glutathione (GSH) quantification; Figure S2: Ramachandran plot assessing the stereochemical quality of the engineered St-GshF protein structure model. Reference [41] is cited in the Supplementary Materials.

## Abbreviations

The following abbreviations are used in this manuscript:

LB	Luria–Bertani
TB	Terrific Broth
GSH	Glutathione
PBS	Phosphate-buffered saline
Tris-HCl	Tris(hydroxymethyl)aminomethane hydrochloride
IPTG	Isopropyl β-d-1-thiogalactopyranoside
MMseqs2	Many-against-Many sequence searching
PAML-X	Phylogenetic Analysis by Maximum Likelihood, X version
DTNB	Ellman’s Reagent Method
HPLC	High-Performance Liquid Chromatography
MD	Molecular dynamics
GshF	Bifunctional Glutathione Synthase
St-GshF	GshF from Streptococcus thermophilus
Anc427	The node 427 ancestral enzyme
SDS-PAGE	Sodium dodecyl sulfate–polyacrylamide gel electrophoresis
Tm	Thermal denaturation temperature
T1/2	Half-life
ASR	Ancestral sequence reconstruction
RMSD	Root-mean-square deviation
AlphaFold2	A deep learning–based protein structure prediction algorithm developed by DeepMind
SAVES v6.1	Structure Analysis and Verification Server, UCLA, USA
AKTA pure	Automated fast protein liquid chromatography system (Cytiva, Uppsala, Sweden)
