# Supplementary material for: Ancestral Sequence Reconstruction for Novel Bifunctional Glutathione Synthetase with Enhanced Thermostability and Catalytic Efficiency
Source: Foods. 2026 Jan 15;15(2):309. doi: 10.3390/foods15020309 (PMC12840475; doi:10.3390/foods15020309)
Supplement: Supplementary file 1 [file foods-15-00309-s001.zip › foods-4066544-supplementary.pdf]

Supplementary Materials

Ancestral Sequence Reconstruction for novel Bifunctional Glutathione Synthetase with Enhanced Thermostability and Catalytic Efficiency

Jieru Zhao [1],Binghao Wang [1,2], Junhua Di [1], Jieyu Zhou [1], Jinjun Dong [1], Ye Ni [1*] and Ruizhi Han [1*]

1 Key Laboratory of Industrial Biotechnology, Ministry of Education, School of Biotechnology, Jiangnan University, Wuxi 214122, Jiangsu, China, zjr20211030@163.com, 7240201048@stu.jiangnan.edu.cn

2 Institute of Biotechnology, RWTH Aachen University, Aachen 52074, Germany

* Correspondence: hanrz@jiangnan.edu.cn, yni@jiangnan.edu.cn

Table S1. Reaction system for glutathione (GSH) determination using the DTNB method.

Component	Final Concentration	Solvent/Buffer	Volume (μL)	Notes
Sample Solution	-	-	6	-
NaOH	0.15 M	-	18	-
Formaldehyde	3% (v/v)	-	6	Let the reaction stand for 2 minutes
DTNB Working Solution	0.1 mM	0.25 M Tris-HCl (pH 8.5)	150	Freshly prepared from 1 mM stock
Total Volume	-	-	180	Reaction at 25°C for 5min

Table S2. Sequence identity comparison between the reconstructed ancestral enzymes and the probe enzyme (St-GshF). The top 5 aligned sequences are listed.

Ancestral enzyme node	Per. ident
427	79.17
428	61.65
426	50.94
429	50.5
430	50.07

Table S3. The amino acid sequence of Anc427.

Ancestral enzyme	Amino acid sequence
**Anc427**	MTINQLLQKLDAASPILQATFGLERESLRVNQEGRVAQTPHPSSLGSRSFHPYIQTDFSE PQLELITPVAHSTKEARRFLGAITDVAGRSIDKDEHLWPLSMPPQLTEDEIQIAQLEND YERHYRQGLAEKYGKKL QAISGIHYNMELGKDLVQALFQASNYSSFKAFKNDLYLKL AQNFLRYRWFLTYLYGAAPLAEKGFYDEEVSQPVRSFRNSDYGYVNDENIQVSFASLE QYVTDIENYVESGELSAEKEFYSAVRFRGQKHNRDYLEQGITYLEFRCFDLNPFDHLG ISQETLDTVHLFLLALLWLDSPEDVDQALQQAHELNDKIALSHPLEPLPAEADTSAILT AMEAVIQHFQLPDYYQQLLDQVKAALTDPQLTLSGQLLPHIENHSLAAFGLEKAQEY HDYAWTAPYALKGYENMELSTQMLLFDAIQKGVHFEILDENDQFLKLWHGQHVEYV KNGNMTSKDNYVIPLAMANKTVTKKILAAAGFPVPAGAEFSSLEEGLAYYPLIKDKIVV KPKSTNFGLGISIFQEPASLESYQKALEIAFAEDSAVLVEEFIAGTEYRFFVLDGKCEA VLLRVAANVVGDGQHTVRELVALKNDNPLRGRDHRSPLEIIELGDIELLMLDQQGYT PDDILPAGVKVDLRRNSNISTGGDSIDVTETMHPSYKELAADMAKAMGAWACG VDL IIPDSTLPSTKENPNCTCIELNFNPSMYMHTYCAEGPGQSITPKILAKLFPEID

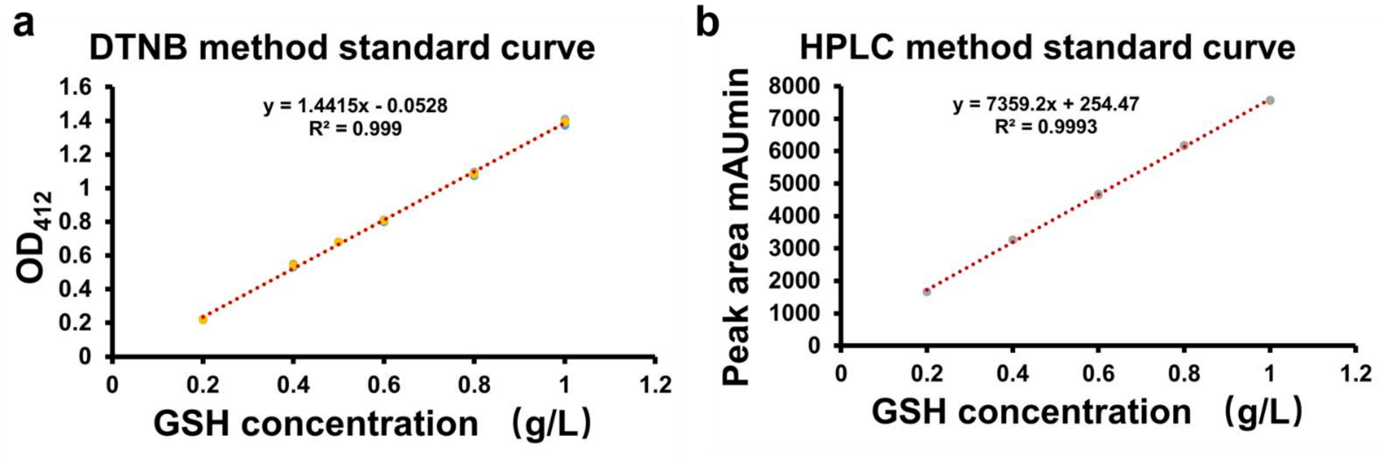

Figure S1: Standard curve for reduced glutathione (GSH) quantification. (a) Standard curve for reduced gluta-thione (GSH) quantification by the DTNB method. (b) Standard curve for reduced glutathione (GSH) quantification by the HPLC method.

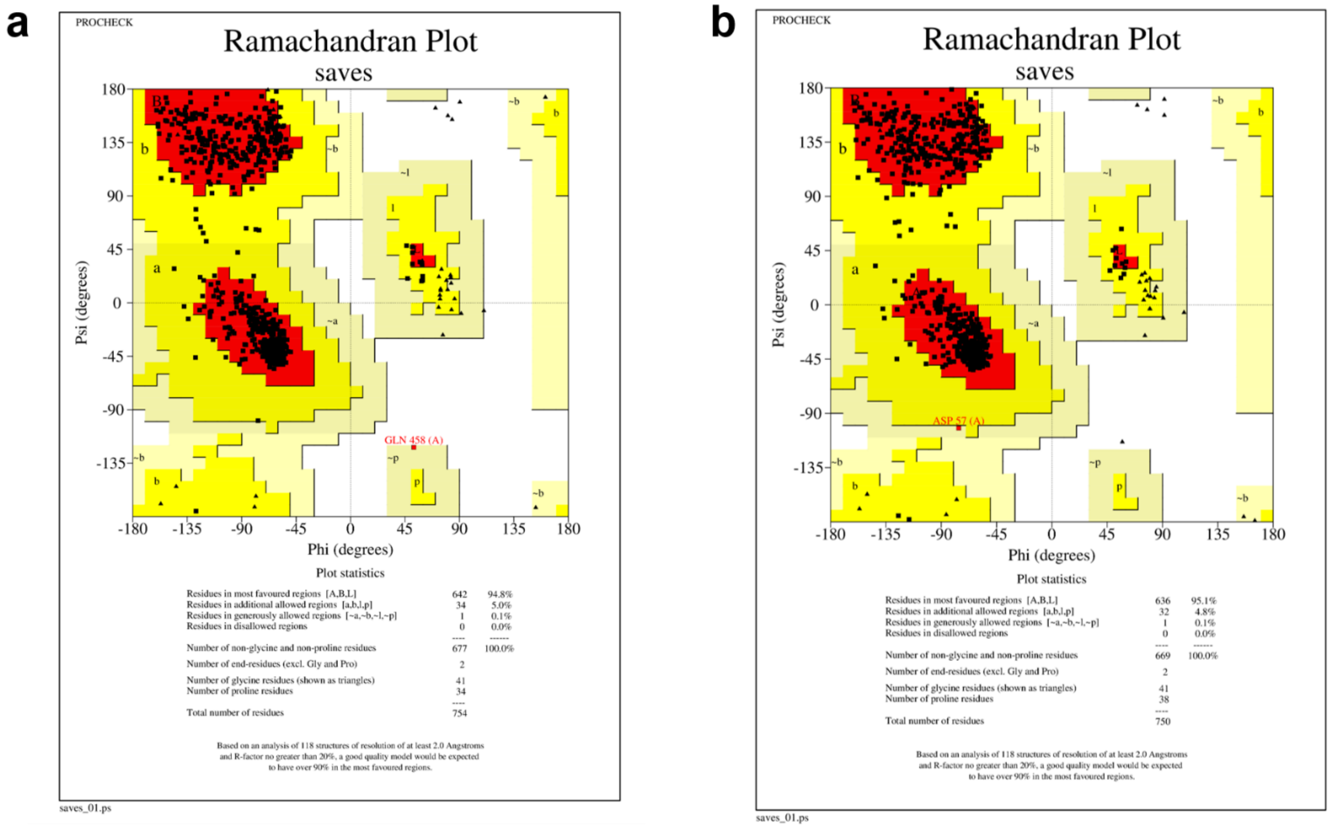

Figure S2. Ramachandran plot assessing the stereochemical quality of the engineered St-GshF protein structure model. (a) St-GshF; (b)Anc427. The phi (Φ) and psi (Ψ) torsion angles of all amino acid residues are plotted. The most favored, additionally allowed, generously allowed, and disallowed regions are indicated in dark red, light red, yellow, and white, respectively. The distribution shows that over 99% of residues fall within the allowed regions, indicating a stereochemically favorable and high-quality model **Error! Reference source not found.**.

References

[1] Laskowski, R.A., Rullmann, J.A.C., MacArthur, M.W. *et al.* AQUA and PROCHECK-NMR: Programs for checking the quality of protein structures solved by NMR. *J Biomol NMR*,**1996**,**8**, 477–486.
